# Radical Response: Effects of Heat Stress-Induced Oxidative Stress on Lipid Metabolism in the Avian Liver

**DOI:** 10.3390/antiox10010035

**Published:** 2020-12-30

**Authors:** Nima K. Emami, Usuk Jung, Brynn Voy, Sami Dridi

**Affiliations:** 1 Center of Excellence for Poultry Science, University of Arkansas, Fayetteville, AR 72701, USA; nkhodamb@uark.edu; 2 College of Arts & Sciences, University of Tennessee, Knoxville, TN 37996, USA; ujung@vols.utk.edu (U.J.); bhvoy@utk.edu (B.V.)

**Keywords:** heat stress, avian, liver, lipid metabolism, oxidative stress

## Abstract

Lipid metabolism in avian species places unique demands on the liver in comparison to most mammals. The avian liver synthesizes the vast majority of fatty acids that provide energy and support cell membrane synthesis throughout the bird. Egg production intensifies demands to the liver as hepatic lipids are needed to create the yolk. The enzymatic reactions that underlie de novo lipogenesis are energetically demanding and require a precise balance of vitamins and cofactors to proceed efficiently. External stressors such as overnutrition or nutrient deficiency can disrupt this balance and compromise the liver’s ability to support metabolic needs. Heat stress is an increasingly prevalent environmental factor that impairs lipid metabolism in the avian liver. The effects of heat stress-induced oxidative stress on hepatic lipid metabolism are of particular concern in modern commercial chickens due to the threat to global poultry production. Chickens are highly vulnerable to heat stress because of their limited capacity to dissipate heat, high metabolic activity, high internal body temperature, and narrow zone of thermal tolerance. Modern lines of both broiler (meat-type) and layer (egg-type) chickens are especially sensitive to heat stress because of the high rates of mitochondrial metabolism. While this oxidative metabolism supports growth and egg production, it also yields oxidative stress that can damage mitochondria, cellular membranes and proteins, making the birds more vulnerable to other stressors in the environment. Studies to date indicate that oxidative and heat stress interact to disrupt hepatic lipid metabolism and compromise performance and well-being in both broilers and layers. The purpose of this review is to summarize the impact of heat stress-induced oxidative stress on lipid metabolism in the avian liver. Recent advances that shed light on molecular mechanisms and potential nutritional/managerial strategies to counteract the negative effects of heat stress-induced oxidative stress to the avian liver are also integrated.

## 1. The Liver and Lipid Metabolism in Avian Species

The avian liver plays a unique role in lipid metabolism in comparison to most mammalian species. Unlike rodents, ruminants and many other mammals, liver is the main site of de novo lipogenesis in avian species. Up to 90% of fatty acids that are synthesized in vivo come from the liver, with relatively minor contributions from adipose tissue [1,2,3,4]. The hepatic contribution of fatty acids is even more significant in some avian species, such as commercial poultry, in which dietary fat content is relatively low [5]. Fatty acids synthesized by the liver are incorporated into triacylglycerol and packaged into very low-density lipoprotein (VLDL) molecules, which ferry this key energy source to other tissues for immediate use or storage. This role makes the avian liver a key player in the regulation of body fat deposition, as most fat stored in adipose tissue originates from hepatic synthesis. The critical role of avian liver in lipid metabolism is also highlighted during egg production, which demands a shift of hepatic lipids to the yolk to nourish the embryo. Under the influence of estrogen, liver synthesizes the protein vitellogenin and a specialized yolk-targeted form of VLDL (VLDLy). This form of VLDL transports triglycerides to the oocyte, but markedly increases demand on lipid synthesis machinery in the avian liver. Therefore, in addition to key roles in metabolism of carbohydrates, proteins, vitamins and minerals, and removal of waste products and detoxification, liver is the central hub for fat metabolism in avian species. This makes the liver an utmost important organ when studying the lipid metabolism in poultry.

Unique anatomical aspects of the circulatory system make the avian liver vulnerable, especially to ectopic lipid deposition and the resulting metabolic stress that it imposes to hepatocytes. In mammals, dietary lipids in the gastrointestinal tract are absorbed into the lymphatic system and carried in chylomicrons before delivery to the circulation [6]. However, birds do not have a well-developed lymphatic system. Instead, fat absorbed in the gastrointestinal tract directly enters the hepatic portal system in the form of portomicrons and reaches the liver [7]. This unique physiological aspect makes avian species that are well-fed, such as domestic poultry, prone to fat accumulation in the liver. Metabolic diseases such as fatty liver hemorrhagic syndrome (FLHS) and fatty liver kidney syndrome (FLKS) are common in poultry especially egg laying hens [8]. Another difference between birds and mammals is that the relative liver size is larger in birds compared to mammals. In general, the relative size of liver compared to body size decreases by age in poultry species.

Various forms of lipids are synthesized de novo by the liver including triglycerides, phospholipids, and cholesterol. These lipids combined with specific proteins (which are also synthesized by the liver) form lipoproteins with different densities. The density of lipoproteins depends on the proportion and type of the lipids and proteins that are incorporated into the lipoprotein structure. Various types of lipoproteins are very low-density lipoprotein (VLDL), low-density lipoprotein (LDL), intermediate-density lipoprotein (IDL), high-density lipoprotein (HDL), and vitellogenin. VLDL mostly consists of triglycerides and apolipoprotein B, while HDL is formed by the incorporation of phospholipids, cholesterol, and apolipoprotein A-1. Under the influence of estrogen, liver synthesizes vitellogenin during the production phase, which is transported via blood to the ovaries where it is incorporated into the egg yolk. Lipid synthesized by the liver is transferred to the adipose tissue via blood in the form of VLDL. Lipolysis of VLDL and IDL with lipoprotein lipase and hepatic lipase will release triglycerides, while LDL is produced as residue. In laying hens, when the production cycle starts, liver size and lipid content increase due to the following: (1) rate of lipoprotein synthesis by the liver is faster compared to their metabolism by hepatocytes; (2) the rate of VLDL release into the bloodstream is greater than their absorption rate by the ovaries, which leads to an increase in the concentration of triglycerides in the blood.

## 2. The Liver as a Target of Stressors

As a crossroads of the body’s metabolic activity, the liver is highly susceptible to external stressors that can disrupt metabolic homeostasis in the liver and throughout the system. The liver’s ability to balance lipid synthesis and utilization depends on efficient mitochondrial metabolism of proteins, fats and carbohydrates. Oxidative metabolism is itself a source of stress, in the form of reactive oxygen species (ROS) that are produced as electrons leak from the respiratory chain during synthesis of ATP. Reactive oxygen species describes a collection of oxygen-based molecules that either contain a free radical that can oxidize cellular components or that are readily converted to a reactive species. These byproducts can oxidatively damage cellular proteins and lipids but are normally kept in check by the liver’s robust system of antioxidants and antioxidative enzymes that quickly neutralize excess ROS to maintain the redox balance [9]. A variety of stressors, such as heat stress and ischemia/reperfusion, can compound the load of ROS that the liver must neutralize by disrupting the mitochondrial membrane proton gradient. The additional burden can overwhelm the liver’s buffering system, resulting in oxidative damage to enzymes, cellular lipids, and the mitochondrial membrane. In addition, ROS are signaling molecules that affect gene expression, therefore, their concentration should be regulated by the antioxidant system [9,10,11].

## 3. Heat Stress Is a Chronic Stressor of Major Concern for Avian Species

Global warming threatens all kinds of life on earth [12], and increased frequency and severity of heat waves are predicted over the next decades [13]. Heat stress occurs when the amount of heat produced by an animal surpasses the animal’s capacity to dissipate the heat to its surrounding environment [14]. Avian species are particularly susceptible to heat stress [15] due to their unique physiology and high metabolic rates. In birds, normal body temperatures are 42 °C compared to 37 °C in mammals, which makes them more susceptible to high ambient temperatures. Unlike mammals, birds do not have sweat glands to promote evaporative cooling and dissipate body heat [16,17,18,19]. In high ambient temperatures (above 28 °C), birds also use panting as a means for evaporative heat loss, but this is only efficient if the humidity is not too high. Instead, birds rely on conduction, radiation and convection of body heat into the environment, but the efficacy of these processes is challenged by the presence of feathers that cover most of the body [17,20].

## 4. Heat Stress Induces Oxidative Stress

**Figure 1:**
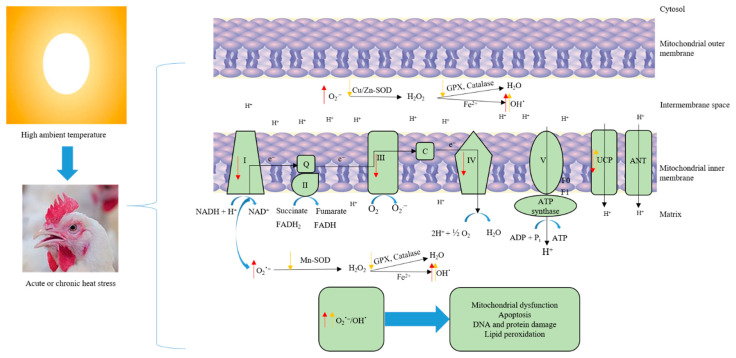
Heat load induces oxidative stress and mitochondrial dysfunction. Acute heat stress suppresses the activity of mitochondrial respiratory complexes I, III, and IV, downregulates uncoupling protein expression, and increases electron leakage, which in turn, leads to increased ROS production (Red arrows). Excessive ROS (O_2_^−^, OH) production causes damage to the protein, lipid, and DNA. Chronic heat stress, on the other hand, reduces the metabolic capacity of mitochondria via downregulating the activity and expression of antioxidant enzymes, and depleting the body’s antioxidant reserves, which causes accumulation of ROS—breaking the oxidative balance and inducing oxidative stress (yellow arrows).

At the cellular level, heat stress is intertwined with oxidative stress through its impact on mitochondrial function. Electrons derived from cellular metabolism are passed along the mitochondrial electron transport chain, generating a proton gradient across the mitochondrial membrane that ultimately drives synthesis of ATP as protons transfer down the electrochemical gradient through ATP synthase. Electrons can also leak back across the membrane and reduce molecular oxygen to produce the highly reactive anion radical superoxide. Low levels of superoxide production are an intrinsic part of cellular respiration and are required to maintain normal redox status of the cell. ROS species at low levels are actually beneficial, as they act as signaling molecules that fine tune various components of cellular metabolism. Healthy cellular ROS levels are maintained by the actions of a system of antioxidant enzymes, including superoxide dismutase (SOD), catalase (CAT), and glutathione peroxidase (GSH-Px), that convert ROS to more inert species (Figure 1) [9]. Superoxide levels are continually buffered by SOD, which converts superoxide to hydrogen peroxide (H_2_O_2_). Peroxide is relatively more stable that superoxide and other ROS species and can diffuse within the cell to oxidatively damage proteins and lipids. Normally, however, H_2_O_2_ levels are kept in check by the actions of CAT, which converts H_2_O_2_ to water and molecular oxygen.

The rate of ROS production is inversely related to the rate of electron transport. Slowing of electron transport chain activity increases the likelihood that electrons will leak back across the membrane before reaching ATP synthase. Under normal conditions, the rate of the electron transport chain is controlled by ATP consumption, with high rates of energy use driving chain activity. Conversely, factors that inhibit ATP consumption or damage components of the electron transport chain slow transport and increase ROS production [21]. Under basal metabolic conditions, ~2–4% of electrons that are transferred to the electron transport chain leak back across prematurely and generate superoxide. However, stress conditions such as heat stress increase electron leakage and subsequently production of ROS [21,22]. The increase in electron leakage and elevated ROS levels during thermal exposure arise in part from suppression of the electron transport chain and ATP synthesis. Acute exposure (3 h) of broiler chickens to heat stress rapidly suppressed activity of mitochondrial respiratory complexes (I, III, and IV) and increased ROS production in the liver (Figure 1). Complexes I and III are the main sites of ROS generation in the electron transport chain. Normally, the cell’s robust anti-oxidant system would mitigate spikes in cellular ROS that are produced by cellular stressors. However, despite a corresponding upregulation of SOD, CAT, and GSH-Px activities, 3 h of heat insult was sufficient to oxidatively damage cellular lipids and proteins, indicating that heat stress overwhelms the capacity of the liver’s buffering system [23]. In addition to indirect ROS production through inhibition of electron transport chain activity, heat stress can also directly increase synthesis of ROS. NADPH oxidase (NOX) proteins are a family of enzymes that transfer electrons across membranes to oxygen, producing the oxygen radical superoxide as a product [24]. Acute heat stress was shown to rapidly (within one hour) upregulate expression of NOX4, a broadly expressed NOX family member, in the liver of broiler chicks, along with parallel increases in expression of SOD and other enzymes that mitigate oxidative stress [25]. 

Mitochondria are both the primary generator of cellular ROS and a highly sensitive target of ROS-mediated damage. Accordingly, compromised mitochondrial function and the subsequent impact on system metabolism and energy balance are primary determinants of the effects of heat stress on the avian liver. The integrity of the mitochondrial membrane is critical in maintaining efficient substrate metabolism. Because of their proximity to the site of ROS generation, phospholipids in the mitochondrial membrane are especially susceptible to oxidative damage. The mitochondrial membrane is enriched in phospholipids containing polyunsaturated fatty acids in their lipid tails. The increased density of double bonds makes polyunsaturated fatty acids highly sensitive to peroxidation by ROS. Oxidative damage to mitochondrial phospholipids produces reactive byproducts such as malondialdehyde (MDA) and 4-hydroxy-trans-2-nonenal (4-HNE) that can contribute to ROS-induced suppression of electron transport chain activity by forming adducts with and inhibiting chain components [26]. The phospholipid cardiolipin is particularly sensitive to peroxidation and oxidative damage that compromises mitochondrial function during oxidative stress. Cardiolipin is an abundant mitochondrial phospholipid that primarily resides in the inner mitochondrial membrane, where it binds and interacts with proteins of the electron transport chain, including cytochrome c [27,28]. Due to the relatively high abundance of polyunsaturated fatty acids in its phospholipid tails, oxidative damage to cardiolipin impairs cellular respiration and contributes to hepatic mitochondrial dysfunction in a range of liver diseases that are exacerbated by oxidative stress, such as the nonalcoholic steatohepatitis (NASH) [29]. Oxidation of cardiolipin was shown to markedly impair activity of Complex I, the initial and rate-limiting component of the electron transport chain [30]. Damage to cardiolipin can also prompt the release of cytochrome C from the mitochondrial membrane into the cytoplasm [31]. Cytochrome C transfers electrons from Complex III to IV and is anchored to the outer surface of the inner mitochondrial membrane by cardiolipin. Release of cytochrome C, which results when cardiolipin is oxidatively damaged and occurs during heat stress, impairs respiratory chain activity, which further increases ROS generation [32]. Cytochrome C release into the cytoplasm can also initiate the intrinsic pathway of apoptosis [33]. These consequences of elevated cellular ROS can initiate a feed-forward chain of reactions that further increase oxidative stress and impair cellular metabolism. Given that the high metabolic activity of the liver and the ability of heat stress to rapidly induce oxidative stress in this tissue, it is likely that oxidative damage to cardiolipin and other members of the mitochondrial membrane play an integral role in the consequences of heat stress in the avian liver. 

Cellular adaptations that buffer or prevent excess ROS levels preserve mitochondrial function and allow tissues to adapt to chronic heat stress. Mitochondrial production of ROS increases as a function of mitochondrial membrane potential. Uncoupling proteins (UCPs) insert into the mitochondrial membrane and upon activation by fatty acids dissipate the membrane proton gradient, which reduces superoxide production [34]. Expression of UCP1, the main UCP in mammals, has been shown to counteract oxidative stress through this mechanism, and expression of UCP is induced by ROS in various cell types. Avian genomes contain a single UCP protein (avUCP) that is orthologous to mammalian UCP3 and primarily expressed in skeletal muscle [35]. Acute heat stress in quail and broilers has been shown to paradoxically decrease expression of avUCP in skeletal muscle, which would exacerbate rather than mitigate ROS synthesis [36]. Similar effects were shown for avUCP in the liver, although expression levels are very low compared to muscle. In the later stages of acute heat stress, excessive ROS causes damage to the protein, lipid, and DNA, which reduces mitochondrial energy production efficiency and increases production of ROS causing mitochondrial dysfunction and increasing the oxidative stress of the body. However, chronic heat stress can reduce the metabolic capacity of mitochondria due to it upregulating the UCPs, downregulating antioxidant enzymes, and depleting the body’s antioxidant reserves, which causes accumulation of ROS—breaking the oxidative balance and inducing oxidative stress [14,37]. 

## 5. Heat Stress and Oxidative Stress in Commercial Poultry—A Perfect Storm

In addition to the physiological features that make avian species in general more susceptible to heat stress, commercial poultry are at even greater risk due to aspects of the production environment and their high growth rates for broilers and high egg production for layers. Intensive poultry production involves raising a large number of birds in a relatively small space. Although most commercial poultry are raised under controlled conditions, maintaining the barn temperature at thermoneutral during the summer is somewhat impossible in many parts of the world. In addition, laying hens that are raised in cages can be easily affected by heat stress as they are unable to seek a cooler place and usually ventilation and air movement is not enough for efficient heat loss through convection. Increased mitochondrial energy generation is intrinsically associated with an increase in ROS production [14]. Commercial lines of poultry, which have been selected for high rates of growth (broilers) or egg production (layers) are particularly susceptible due to their greater metabolic activity, higher heat production, and decreased thermo-tolerance [16,38]. As a result, the thermoneutral zone of most poultry is relatively narrow. A high growth rate in modern broilers has increased the metabolic demand for oxygen due to higher metabolic rate, which in turn increases ROS generation [39,40]. This is particularly true in tissues like liver that have high rates of substrate metabolism and energy expenditure [21]. As a result of these multiple factors that increase susceptibility, heat stress is one of the most important environmental stressors that adversely affects poultry production worldwide by the induction of oxidative stress, thus overproduction of ROS [41,42]. 

Overproduction of free radicals compromises antioxidant defense, and oxidative stress is the leading cause of the detrimental consequences of stress in poultry [9]. The balance between antioxidants and prooxidants (redox balance) in the body controls various processes ranging from homeostasis maintenance to gene expression and cell signaling [43,44]. Heat stress induces the formation of intracellular hydrogen peroxide (H_2_O_2_) and 4-hydroxynonenal (4-HNE), which in turn alter the avian neuroendocrine system, leading to the activation of the HPA stress axis, increased circulating corticosterone levels, and apoptotic cell death [45,46,47,48]. Heat stress could negatively affect nutrient digestion and absorption by increasing the production of ROS and subsequent disruption of antioxidant system [23,49]. The gut plays a crucial role in nutrient digestion and absorption, and morphological alterations of the gut epithelium were observed in heat-stressed poultry [50]. Reduced villus absorptive surface area combined with altered expression of intestinal nutrient transporters negatively affect nutrient absorption. For instance, heat stress has been shown to alter the expression of jejunal glucose transporter 2 (GLUT2), fatty acid binding protein1 (FABP-1), and cluster of differentiation 36 (CD36) [51]. Furthermore, heat stress reduces the activity of digestive enzymes such as amylase, maltase, lipase, trypsin, and chymotrypsin, which in turn results in reduced dietary digestibility [52]. In addition, heat stress induces mucosal lesions in the small intestine and leaky gut syndrome [53,54]. Comparison of low and high feed efficient broilers showed higher production of H_2_O_2_ and protein carbonyls in the liver of low feed efficient broilers [55]. This might be the case in heat stress condition as heat stress negatively impacts feed efficiency [5,54].

## 6. Effects of Heat Stress in Poultry

Across species, heat stress suppresses feed intake in a compensatory effort to reduce endogenous production of body heat that results from digestion and absorption of feed [56,57]. The negative effects of heat stress on feed intake are greater in fast compared to slow growing birds [58]. Heat exposure decreases feed intake by about 3.4% in fast growing broilers and 1.7% in slow growing chickens per degree increase in temperature between 21 and 34 °C [58]. This is a physiologically important phenomenon as the body starts to use its reservoirs as an energy source instead of digestion, absorption and metabolism of carbohydrates, proteins, and lipids from the feed [41]. Feed digestion and absorption produces a great amount of heat and reliance on fat reserves produces less heat which is critical in reducing heat production in times of high ambient temperatures. However, when poultry are exposed to heat stress, their body temperature increases sharply, and to dissipate the excess heat, blood circulation, and peripheral blood flow increases, whereas their visceral blood flow decreases [41]. These changes lead to limited nutrients utilization and thus, reduce the poultry’s production performance and feed conversion efficiency [37,57,59,60].

Despite the reduction in feed intake, heat stress has been shown to increase fat deposition in poultry as well as in other species. Broilers raised at 34 °C were shown to retain 25% more dietary energy as body fat compared to those raised in thermoneutral (22 °C) conditions [61]. Heat stress increases the deposition of fat in adipose tissue, particularly in the abdominal depot [58]. Even a relatively modest duration (7D) of heat stress increased abdominal adiposity by ~20% in comparison to pair-fed controls raised under thermoneutral conditions [62]. Sustaining the period of heat stress further promotes adiposity, with up to an 80% increase in abdominal fat reported in broilers after three weeks of heat stress [58]. As the majority of fat stored in avian adipose tissue comes from VLDL supplied by the liver, aberrant fat deposition in heat-stressed broilers likely results from effects on hepatic lipid synthesis. Consistent with this relationship, heat stress was shown to upregulate rate-limiting components of de novo lipogenesis and increase liver triglyceride content and plasma VLDL levels in broilers [62]. Exposure of broilers to heat stress for 7 d significantly increased the levels of triglycerides and VLDL in plasma, as well as the levels of triglycerides, total cholesterol, acetyl-CoA carboxylase (ACC), and fatty acid synthase (FASN) in the liver, and mRNA expression levels of carbohydrate response element-binding protein (ChREBP), ACC, FASN, and microsomal triglyceride transfer protein (MTTP) (Table 1) [62]. The increase in circulating levels of VLDL, hepatic triglycerides, and FASN protein persisted after fourteen days, suggesting that these responses were part of a stable adaptation to chronic heat stress. Acceleration of lipogenesis under heat stress increases the burden on the hepatic system of lipid delivery system, which relies on efficient synthesis of lipoproteins that are needed to export triglyceride from the liver. During the initial adaptation to heat stress, increased transport of VLDL to adipose tissue appears to match the increased rate of lipid synthesis. However, sustained heat stress may overwhelm the ability of the liver to export sufficient lipids, reflected in significant increases in liver weight and triglyceride content in broilers after 14 d of heat stress [62]. As a result, chronic heat stress may further compromise hepatic function by progressively increasing ectopic lipid deposition and promoting hepatic steatosis [62]. Consistent with this concept, similar levels of apolipoprotein B in the liver were reported for heat-stressed and thermoneutral birds, despite a significant increase in demand for lipid export under heat stress [62].

Compared to birds raised under normal ambient temperature, chronic heat stress (34 °C) significantly reduced the relative weight of the liver in broiler chicks, which might be the result of a reduction in metabolic needs [63,64]. Exposure to chronic heat leads to a significant decrease in total lipid and triglyceride levels in the liver of broiler chicks, while it leads to increased total cholesterol levels compared to the birds raised under normal temperature (22 °C) [64,65,66]. Reduction in total lipid level in the liver of birds exposed to high ambient temperature could be indicative of higher reliance on lipid reservoirs as an energy source during heat stress. In addition, greater C 18:0, and lower C 16:1 and total unsaturated fatty acid content has been reported in the liver of birds exposed to high (34 °C) ambient temperature [64], which might be another indicator for greater reliance on de novo fatty acid metabolism during heat stress due to reduced feed intake. On the contrary, chronic heat stress (32 °C) increased triglyceride levels in the liver compared to birds raised at normal temperature [62]. Discrepancy between the results might be due to the duration and severity of the heat stress, species and sex of the bird, and age at which heat stress is induced.

At the organismal level, heat stress causes several physiological changes. In addition to oxidative stress, heat stress induces acid-base imbalance, respiratory alkalosis, immune-competence suppression, feed intake depression, body weight and egg production reduction, meat and egg quality alteration, and in severe cases increased mortality by spiraling hyperthermia; as a result, it inflicts heavy economic losses upon the industry [67].

## 7. Molecular Response to Heat Stress in the Avian Liver

Genome-wide transcriptomic studies have identified numerous pathways that are altered by acute and chronic heat stress in the liver of broiler chickens. The effects of a summer heat wave were mimicked by exposing broiler chicks to heat stress daily (8 h per day) for one week. This relatively short period of cyclical heat stress altered the expression of 1299 genes in the liver compared to chicks maintained under thermoneutral conditions. Pathway analyses highlighted a broad range of affected cellular responses, including processes related to apoptosis, cell cycle, DNA repair, membrane trafficking, and immune function (Table 1) [68]. Genes involved in lipid metabolism were highly enriched in the set of genes induced by heat stress. Several genes encoding for enzymes at various phases of fatty acid synthesis were also elevated due to the heat stress. In particular, expression of acetyl-CoA carboxylase alpha (ACCα), which carboxylates Acetyl-CoA to malonyl-CoA for the first step in synthesis of palmitate, was significantly increased by heat stress [68], consistent with increased hepatic triglyceride synthesis in heat-stressed chicken described by others [62]. Expression of stearoyl-CoA-desaturase-1 (SCD-1), which catalyzes the desaturation of palmitate (16:0) and stearate (18:0), was also upregulated by heat stress. These authors also integrated metabolomics with transcriptomics to more thoroughly profile the impact of heat stress on hepatic metabolism. Liver content of glycerol and glycerol-3-phosphate were elevated compared to thermoneutral controls, which may have reflected increased demand through glyceroneogenesis for the backbone used in triglyceride synthesis [68]. In combination with gene-focused studies that have also described upregulation of key lipogenic genes in heat-stressed liver [5], these findings clearly demonstrate that heat stress transcriptionally regulates lipogenesis in the avian liver. 

Transcriptomics has also provided additional insight into the acute response to heat stress in the avian liver [69]. Three hours of an acute heat stress was sufficient to alter expression of 627 genes in broiler liver compared to thermoneutral controls. Gene Ontology analyses identified a broad set of pathways affected by acute heat stress, including functions related to ion channel activity, redox balance, and lipid metabolism. Network analyses highlighted predicted interactions between many of these genes and heat shock proteins (e.g., Hsp70, Hsp90) that coordinate the initial response to thermal stress [69]. Heat shock proteins are known for their role as chaperones that maintain cellular integrity during stress by identifying and trafficking misfolded or damaged proteins into degradation. Several members of this gene family have been shown to be induced by heat stress in chickens, including Hsp70. Notably, overexpression of Hsp70 has been shown to promote lipogenesis in a liver cell line (HepG2) [70]. It is therefore intriguing to speculate that induction of Hsp70 may contribute to the upregulation of fatty acid synthesis and triglyceride accumulation that occur in the liver of heat-stressed chickens. Acute heat stress also induced a large fold-change in expression of NADPH oxidase 1 (NOX1). NOX1 and other NADPH oxidase family members generate superoxide and other ROS by transporting electrons across cell membranes [24]. Induction of NOX1 expression may therefore contribute to generation of oxidative stress in the liver during the initial stage of heat stress. 

## 8. Dietary Interventions to Alleviate the Negative Effects of Heat Stress on Lipid Metabolism

Various dietary modifications have been used to alleviate the negative impact of heat stress on birds [74]. Most of these studies on the protective roles of additives in heat stress are focused on dietary supplementation of products with antioxidant activity such as phytochemicals, trace minerals, and antioxidants. Our approach here is to summarize the results of these studies.

Concentration of ascorbic acid in the serum and liver decreases during heat stress; thus, ascorbic acid supplementation to quails and laying hens under heat stress reduced MDA in the serum and liver [74,75,76]. Supplementation of vitamin E alone or in combination with ascorbic acid decreased MDA and TBA concentration in the serum of laying hens [75,77].

Dietary supplementation of taurine (0.8%) alleviated the negative impact of chronic heat stress (34 °C) and increased the relative weight of the liver in broiler chicks [64]. In addition, dietary taurine supplementation under the heat stress condition resulted in recovery, to the control group levels (birds raised in normal temperature), of serum triglyceride concentrations, total lipids, triglycerides, and cholesterol, as well as the amounts of C 18:0, C 16:1 and total unsaturated fatty acids in the liver [64].

Betaine, due to its osmoregulatory functions, and ability to improve the activity of the superoxide dismutase (SOD) and glutathione peroxide (GPx) enzymes in heat-stressed birds plays an important role in alleviating heat stress in poultry [78,79,80]. Dietary supplementation of betaine decreased MDA content, and increased SOD and GPX activity in the breast muscle of birds exposed to heat stress compared to heat-stressed birds fed the control diet [81].

Supplementation of glutamine significantly increased serum glutathione, total superoxide dismutase activity, and total antioxidant capacity and reduced MDA in birds exposed to chronic heat stress compared to birds fed control un-supplemented diet [82].

Administration of amino acid-chelated trace minerals (containing Zn, Mn, Fe, Cu, Co, Mg, K and Ca) to drinking water of broilers alleviated the negative impact of chronic heat stress and decreased the expression of HSP70, IL-18, TNF-α, and NLRP3 in the blood [83]. 

Phytochemicals are a particularly promising toolset with which to combat the effects of heat stress in poultry. Because of the general relevance of oxidative stress to human health and aging, the list of known phytochemicals and bioactive compounds that have been shown to combat ROS continues to grow, and many have been tested for their protective effect against heat stress. Resveratrol is a polyphenolic compound found in grapes and berries that has potent antioxidant activity. Resveratrol works in part by inducing activity of Nrf-2, a transcription factor that in turn induces expression of SOD, CAT and other anti-oxidants [84]. Dietary supplementation of resveratrol in rats was shown to alleviate liver damage induced by heat stress. Resveratrol can inhibit lipid peroxidation and significantly improve antioxidant enzyme (SOD, GSH-Px, CAT) activity in hepatocytes [85]. Epigallocatechin-3-gallate (green tea), lycopene (tomato), and resveratrol (red grapes, peanuts and berries) elicit antioxidant effects through inhibiting NF-κB expression and activating Nrf2 expression, which were activated and suppressed in the heat stress environment [86].

Curcumin is another polyphenol with potent anti-oxidant and anti-inflammatory activity that has been examined for its effects under heat stress. Curcumin decreased ROS production but increased mitochondrial membrane potential in hepatocytes of broilers subjected to heat stress [87]. Curcumin mitigated mitochondrial dysfunction in heat-stressed broilers, as evidenced by the suppression of the ROS burst, maintenance of the thiol pool and mitochondrial DNA content, and enhanced mitochondrial antioxidant gene expression (MnSOD and thioredoxin reductase 2) [87]. Curcumin can restore the impaired growth performance caused by heat stress, potentially due to its capacity to mitigate mitochondrial dysfunction caused by heat stress via enhancing the mitochondrial biogenesis [37].

Olive oil is enriched in oleic acid (18:1) and has been shown to have anti-inflammatory and anti-oxidant properties when included in the diet. Supplementing the broiler diet with olive oil was shown to protect broiler skeletal muscle from oxidative stress induced by acute heat stress [88]. Protection was associated with upregulation of avUCP, which is consistent with the benefits of dissipating the mitochondrial proton gradient to reduce ROS synthesis [34]. 

At the organismal levels, and by strengthening the cellular defense against oxidative substances and minimizing DNA and organelle damages, the abovementioned strategies appeared to be potential and promising tools to, at least partially, counteract and alleviate heat stress productivity loss. By improving the antioxidant system, these strategies improve health status of birds, enhance gut integrity, increase feed intake and overall growth performance and bird well-being. 

## 9. Conclusions and Perspectives

The liver plays a crucial role in all metabolic processes. In fat metabolism, the liver is extremely active in oxidizing triglycerides to produce energy. It is a main site for lipoprotein, cholesterol, phospholipids, and de novo fatty acid synthesis. In avian species, more than 95% of lipogenesis occurs in the liver, which is very sensitive to stress, including heat load. Heat stress is one of the most challenging stressors to poultry due to its adverse effects on production, welfare, and mortality. Although seminal work has been conducted to define the mechanism by which heat stress affects avian hepatic lipid metabolism, further mechanistic and functional studies are warranted. The development of different omics approaches will have the potential to identify key molecular signatures for the subsequent development of effective strategies to address heat tolerance in avian species.

## Figures and Tables

**Table 1:** Comparisons of liver transcriptomes in chicken exposed to acute or chronic heat stress.

Ref.	Animal	Heat Stress Condition	Gene Ontology Terms	Pathways	Differentially Expressed Genes ^1^
[69]	Male broiler (17−day−old)	**Control** (25 °C for 7 d) **Acute HS** (35 °C for 3 h) **Chronic HS** (7 h/d for 7 d at 35 °C)	**Under Acute HS** system/tissue/cellular development, response to gonadotropin, cell differentiation, cellular lipid metabolic process, lipid metabolic process, growth factor binding, insulin−like growth factor, oxidoreductase activity−aldehyde, transferase activity **Under Chronic HS** contractile fiber part, extracellular region/matrix, integral/intrinsic component of organelle membrane, myofibril, negative regulation of transmembrane receptor protein, lipid homeostasis, skeletal system, biological adhesion, cell adhesion	**Under Acute HS** cardiovascular system (nitric oxide signaling in the cardiovascular system and cardiac β−adrenergic signaling), nervous system (glioma signaling), immune system (PTEN signaling and NF-kB signaling), hepatic system (HGF signaling and Leptin signaling in obesity), circulatory system (eNOS signaling)	**Under Acute HS** ANGPTL4, NOG, UNC5A, HKDC1, TMEM154, SPINW, TNF, HSP70, HSPA2, DNAJC12, HDAC **Under chronic HS** FST, SIK1, HKDC1, gadd45
[68]	Male broiler (21−day−old)	**Control** (23–25 °C for 7 d) **Chronic HS** (8 h/d for 7d at 35−37 °C)	**Under Chronic HS** cell cycle, DNA repair, immune function, lipid catabolism	circadian rhythm, peripheral liver clock, thyroid hormone signaling, glycogenolysis/gluconeogenesis, fatty acid metabolism, glycosylation	circadian rhythm response (↑: PER2, PER3, PROK2, TEF, PAR bZIP TEF, BHLHE41, CRY) peripheral liver clock (↓: NPAS2, ARNTL) thyroid hormone signaling (↓: DIO2) glycogenolysis/gluconeogenesis (↑: ADCY9, PGM1, FBP2, GPI, SLC2A2) fatty acid metabolism (↑: ACACA, ACSF3) glycosylation (↑: PMM1, PMM2)
[71]	Male broiler (22−day−old)	**Control** (25 °C for 7 d) **Chronic HS** (7 h/d for 7 d at 35 °C)	**Under Chronic HS** cell signaling, endocrine system development and function, molecular transport, small molecular biochemistry, vitamin and mineral metabolism, protein synthesis, cardiovascular system development and function, organ morphology, tissue morphology, cell morphology, cellular development, organismal development, amino acid metabolism, connective tissue disorders, inflammatory disease, skeletal and muscular disorders, auditory and vestibular system development and function, behavior	cell signaling (cellular CCK signaling, apoptosis pathway, calcium signaling), endocrine system development and function (thyroid hormone signaling, blood vessel development, tissue repair response)	cellular CCK signaling (↑: CCK, DIO3, ↓: TRPC5, DIO2) apoptosis pathway (↑: ORMDL3, ↓: SCN3B, SPON1, MYRIP) calcium signaling (↑: ERC2, S100A1, BDKRB1, ↓: S100A4) blood vessel development (↑: BRCC3, FGF14, ↓: ANGPTL4) tissue repair response (↓: FMOD, GPR133, TRIM50)
[72]	Male Broiler (70−day−old)	**Control** (25 °C) **Acute HS** (38 °C for 4 h) **Recovery** (38 °C for 4 h and 25 °C for 2 h)	**Under Acute HS** metabolic progress, single−organism metabolic progress, small molecule metabolic progress, lipid metabolic progress, fatty acid metabolic progress, organic acid catabolic process, carboxylic acid catabolic process, cellular amino acid catabolic process, organic acid biosynthetic process, carboxylic acid biosynthetic process, small molecule biosynthetic process, oxidation−reduction process	fatty acid metabolism, biosynthesis of unsaturated fatty acids, steroid biosynthesis, caffeine metabolism	**Under Acute HS** fatty acid metabolism (ACOX1, ACACA, ACSL1, ACSL6, ACAA1, ACAA2, HADHB, FASN), pyruvate metabolism (ACSS2, ALDH2, ACACA, DLAT, ALDH7A1, MDH1, ME1), propanoate metabolism (ACSS2, ACACA, ABAT, SUCLG2, ACSS3)
[73]	Male Broiler (22−day−old)	**Control** (25 °C) **Chronic HS** (8 h/d for 7 d at 38 °C)	**Under Chronic HS** chromosome segregation, mitotic nuclear division, cell division, condensed chromosome kinetochore, nucleus, chromosome, centromeric region, mid-body, kinetochore, centrosome, ATP binding	Fanconi anemia pathway, cell cycle pathway	**Under Chronic HS** fanconi anemia pathway (FANCA, FANCG, FANCI, PMS2, APITD1, BRCA1, BRCA2, EME1, POLN, UBE2T), cell cycle pathway (BUB1, E2F1, MAD2L1, TTK, WEE1, CDC20, CDC45, CCNA2, CCNB2, CDK1, CDKN2C, MCM6, PTTG1, PLK1)

^1^ ANGPTL4, angiopoietin−like 4; NOG, noggin precursor; UNC5A, unc−5 homolog A; HKDC1, hexokinase domain containing 1; TMEM154, transmembrane protein 154; SPINW, spindling 1; TNF, tumor necrosis factor; HSP70, heat shock protein 70; HSPA2, heat shock protein family A member 2; DNAJC12, DnaJ heat shock protein family member C12; HDAC, histone deacetylase; FST, follistatin; SIK1, serine/threonine−protein kinase SIK2; HKDC1, hexokinase domain containing 1; gadd45; growth arrest and DNA damage−inducible protein GADD45 gamma; PER2, period circadian clock 2; PER3, period circadian clock 3; PROK2, prokineticin 2; TEF, transcription factor; PAR bZIP TEF, PAR bZIP transcription factor; BHLHE41, basic helix−loop−helix family member e41; CRY, cryptochrome; ADCY9, adenylyl cyclase 9; PGM1, phosphoglucomutase; FBP2, fructose−bisphosphatase 2; GPI, glucose−6−phosphate isomerase; SLC2A2, facilitated glucose transporter solute carrier family 2 member 2; ACACA, acetyl−CoA carboxylase alpha; ACSF3, acyl−CoA synthetase; PMM1, phosphomannomutase 1; PMM2, phosphomannomutase 2; NPAS2, neuronal PAD domain protein 2; ARNTL, aryl hydrocarbon receptor nuclear translocator−like; DIO2, deiodinase Iodothyronine Type II; ERC2, ELKS/RAB6−interacting/CAST family member 2; FGF14, fibroblast growth factor 14; ORMDL3, ORM1−like 3; BDKRB1, bradykinin receptor B1; CCK, cholecystokinin; DIO3, iodothyronine, type III; MYRIP, myosin VIIA and Rab interacting protein; S100A1, S100 calcium binding protein A1; BRCC3, BRCA1/BRCA2−containing complex, subunit 3; FMOD, fibromodulin; GPR133, G protein−coupled receptor 133; SCN3B, sodium channel, voltage−gated, type III, beta subunit; SPON1, spondin 1, extracellular matrix protein; TRIM50, tripartite motif containing 50; S100A4, S100 calcium binding protein A4; TRPC5, transient receptor potential cation channel, subfamily C, member 5; ACOX1, acyl−CoA oxidase 1; ACSL1, acyl−CoA synthetase−1; ACSL6, acyl−CoA synthetase−6; ACAA1, acetyl−CoA acyltransferase 1; ACAA2, acetyl−CoA acyltransferase 2; HADHB, trifunctional enzyme subunit beta, mitochondrial; FASN, fatty acid synthase; ACSS2, acyl−CoA synthetase short chain family member 2, ALDH2, Aldehyde dehydrogenase, mitochondrial; DLAT, dihydrolipoamide acetyltransferase; ALDH7A1, aldehyde dehydrogenase 7A1, MDH1, malate dehydrogenase 1; ME1, malic enzyme 1, ABAT, 4−aminobutyrate aminotransferase; SUCLG2, succinyl−CoA ligase subunit beta, mitochondrial; ACSS3, acyl−CoA synthetase short chain family member 3; FANCA, fanconi anemia complementation group A; FANCG, fanconi anemia complementation group G; FANCI, fanconi anemia complementation group I; PMS2, PMS1 homolog 2; APITD1, centromere protein S; BRCA1, breast cancer 1; BRCA2, breast cancer 2; EME1, essential meiotic structure−specific endonuclease 1; POLN, polymerase (DNA) Nu; UBE2T, ubiquitin conjugating enzyme E2 T; BUB1, BUB1 mitotic checkpoint serine/threonine kinase; E2F1, E2F transcription factor 1; MAD2L1, MAD2 mitotic arrest deficient−like 1; TTK, TTK protein kinase; WEE1, WEE1 G2 checkpoint kinase; CDC20, cell division cycle 20; CDC45, cell division cycle 45; CCNA2, cyclin A2; CCNB2, cyclin B1; CDK1, cyclin−dependent kinase 1; CDKN2C, cyclin−dependent kinase inhibitor 2C; MCM6, minichromosome maintenance complex component 6; PTTG1, pituitary tumor−transforming 2; PLK1, Polo like kinase 1.
